# Improved Production of Xylanase in *Pichia pastoris* and Its Application in Xylose Production From Xylan

**DOI:** 10.3389/fbioe.2021.690702

**Published:** 2021-08-27

**Authors:** Ting Miao, Abdul Basit, Junquan Liu, Fengzhen Zheng, Kashif Rahim, Huiqiang Lou, Wei Jiang

**Affiliations:** ^1^ State Key Laboratory of Agro-Biotechnology, College of Biological Sciences, China Agricultural University, Beijing, China; ^2^ Department of Microbiology and Molecular Genetics, Faculty of Life Sciences, University of Okara, Okara, Pakistan; ^3^ Department of Microbiology, Cholistan University of Veterinary and Animal Sciences (CUVAS), Bahawalpur, Pakistan

**Keywords:** *Myceliophthora thermophila*, *Pichia pastoris*, xylanase, improved production, hydrolytic activity, xylan, xylose

## Abstract

Xylanases with high specific activity has been focused with great interest as a useful enzyme in biomass utilization. The production of recombinant GH11 xylanase (MYCTH_56237) from *Myceliophthora thermophila* has been improved through N-terminal signal peptide engineering in *P. pastoris*. The production of newly recombinant xylanase (termed Mtxyn11C) was improved from 442.53 to 490.7 U/mL, through a replacement of α-factor signal peptide with the native xylanase signal peptide segment (MVSVKAVLLLGAAGTTLA) in *P. pastoris*. Scaling up of Mtxyn11C production in a 7.5 L fermentor was improved to the maximal production rate of 2503 U/mL. In this study, the degradation efficiency of Mtxyn11C was further examined. Analysis of the hydrolytic mode of action towards the birchwood xylan (BWX) revealed that Mtxyn11C was clearly more effective than commercial xylanase and degrades xylan into xylooligosaccharides (xylobiose, xylotriose, xylotetraose). More importantly, Mtxyn11C in combination with a single multifunctional xylanolytic enzyme, improved the hydrolysis of BWX into single xylose by 40%. Altogether, this study provided strategies for improved production of xylanase together with rapid conversion of xylose from BWX, which provides sustainable, cost-effective and environmental friendly approaches to produce xylose/XOSs for biomass energy or biofuels production.

## Introduction

Xylanases an integral group of hemicelluloses and offers an opportunity of best interest area for researchers on account of their insightful investigation and prospective of industrial applications, especially in the degradation of biomass into fermentable sugars (Basit et al., 2018a). On account of the heterogeneity and complexity, the complete degradation of xylan necessitates multiple enzymes (particularly endoxylanases and β-xylosidase) in association with accessory enzymes to interplay in the process, which liberates xylooligosaccharides (XOS) (Gaurav et al., 2017). XOS have been extensively applied in a variety of industrial processes, particularly in chemical industries and biofuel production (Samanta et al., 2012).

Albeit, the enzymatic hydrolysis demonstrates better results and low process energy requirements, however, the high cost of commercial enzymes and probably enzymes with low activity, creates a bottleneck for complete degradation of hemicelluloses. Therefore, more potent and efficient enzymes with high activity need to be developed for more economical purposes. Several approaches have been adopted to minimize the expenses of hydrolysis process such as improving enzyme activity and enzyme production (Lin et al., 2017; Zhou et al., 2015; Wang et al., 2012). The genetic engineering paved the way for the mass scale production of xylanases (Uday et al., 2016). Recent developments in recombinant protein engineering also helped in expressing xylanases in heterologous hosts (*Pichia pastoris*) for mass production.

*P. pastoris* has been substantiated as a more successful system for recombinant proteins, due to the high cell densities and therefore yield high volumetric products (Ahmad et al., 2014). Moreover, *P. pastoris* secretes less native proteins and enable the purification of recombinant protein in supernatant easier, through directing signal peptide. To intensify heterologous protein expression in *P*. *pastoris*, different procedures have been established, including the optimization of the secretory signal peptide sequence (Zheng et al., 2018). Protein expression levels have been improved with greater success prospects through regulation of the several factors such as recruitment of natural pro-peptides and gene dosage (Aw et al., 2018). Secretory signal peptides have been assessed to improve the protein expression level in *P. pastoris*, particularly, the secretion/releasing efficiency and optimization of the signal peptide codons of the α-factor. Multiple revealed studies indicate the impacts of secretory signal peptides on the yield expression. Bovine-casein (signal peptide) resulted in lower yield for the secretion of the xylanase *xynB* instead of the α-mating factor (α-MF) peptide from *S. cerevisiae* (He et al., 2012). Similarly, for the expression of bovine pancreatic trypsin inhibitor (BPTI) displayed no effective impact while using the α-MF, however, using human serum albumin (HSA) signal peptide sequence demonstrated an effective secretion into the medium (Yang et al., 2008).

In our previous study, the gene encoding GH11 xylanase MYCTH_56237 from *M. thermophila* was expressed in *P. pastoris* and achieved a high specific xylanase activity of 1533.71 U/mg (Basit et al., 2018b). However, the xylanase secretions into the culture medium was inadequate. Therefore, the present study was aimed to improve the xylanase production through the engineering of N-terminal peptide in *P. pastoris*. As expected an increased production of the xylanase in (both shake flasks and 7.5 L fermentor) was achieved by substituting of the secretory α-MF with the native signal peptide. In this study, the degradation efficiency of newly improved xylanase was clearly more effective and produced xylobiose, xylotriose and xylotetrose from birchwood-xylan (BWX). This study also elucidated the improved conversion of xylan into xylose by 40%, when integrated with the multifunctional xylanloytic enzyme (Basit et al., 2019).

## Materials and Methods

### Reagents

Restriction enzymes and ligases were purchased from New England Biolabs (NEB). Substrates 4-nitrophenyl β-D-xylopyranoside (pNPX), 4-nitrophenyl β-D-glucopyranoside (pNPG), 4-nitrophenyl β-D-cellobioside (pNPC), and commercial xylanase from Sigma-Aldrich (United States). Birchwood-xylan (BWX), commercial β-xylosidase (EC 3.2.1.37, *Bacillus pumilus*), and standard xylooligosaccharides (X_2_, xylobiose; X_3_, xylotriose; X_4_, xylotetraose; X_5_, xylopentaose) were from Megazyme (Ireland). XOSs (a mixture of X_2_, X_3_, X_4_ and X_5_) were provided by Aladdin (X140487), China. X_2_, X_3_, X_4_ and X_5_ were further identified on HPLC analysis.

Cultivation media [Yeast extract peptone dextrose (YPD) and buffered minimal glycerol medium (BMGY)], and gene expression medium for *P. pastoris* [buffered minimal methanol medium (BMMY)] were prepared according to the manual in the *Pichia* Expression Kit (Invitrogen). Other chemicals used were analytical grade unless otherwise stated.

### Construction of Recombinant Plasmids and Strains

The full-length of xylanase gene *MYCTH_56237* was amplified from pMD19-T simple vector harboring the MYCTH_56237 using specific primers (Supplementary Table S1). The amplified PCR product was purified from gel using the Gel Extraction Kit (Thermo Fisher Scientific, Waltham, MA, United States) and ligated to expression plasmid pPICZαA, pre-digested with *Eco*RI and *Xba*I. The resultant recombinant plasmid was transformed into *E. coli* strain DH5α, as further confirmed by PCR and DNA sequencing. The expression plasmid pPICZαA encoding α-factor signal peptide and *MYCTH_56237* were designed to facilitate the construction of variants as described by Zheng et al. (2018).

**FIGURE 1:**
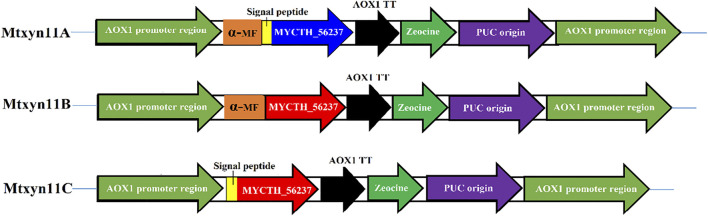
Schematic representation for the construction of plasmid (pPICZαA) having different signal peptides. Mtxyn11A: pPICZαA containing both native signal peptide and α-MF; Mtxyn11B: pPICZαA without native signal peptide; Mtxyn11C: pPICZαA without α-MF.

Enzyme activity of MYCTH_56237 was improved through replacement of α-factor in *P. pastoris* with different signal peptide sequences. The peptide signals of MYCTH_56237 secreted 18 amino acids (MVSVKAVLLLGAAGTTLA) was predicted based on SignalP (http://www.cbs.dtu.dk/services/SignalP/). The native signal peptide segment of MYCTH_56237 positioned between the C-terminus of the α-MF and the N-terminus of pPICZαA termed Mtxyn11A, and pPICZαA without native signal peptide of MYCTH_56237 termed Mtxyn11B. Similarly, the pPICZαA with native signal peptide segment of MYCTH_56237 (without α-MF) termed Mtxyn11C (Figure 1). The expression plasmids Mtxyn11A, Mtxyn11B and Mtxyn11C were constructed with corresponding primers (Mtxyn11A*-*F*/* Mtxyn11A*-*R, Mtxyn11B-F/ Mtxyn11B*-*R and Mtxyn11C*-*F/ Mtxyn11C*-*R) by one step PCR according to the Phanta Max Super-Fidelity DNA Polymerase (primers are listed in the Supplementary Table S1). The recombinant strains Mtxyn11A, Mtxyn11B and Mtxyn11C were obtained by transformation of the corresponding plasmids into *P. pastoris*. Transformants were grown on YPD agar plates containing 100 μg/mL Zeocin (Invitrogen, United States) and further confirmed by colony PCR with AOX-F/AOX-R primers and DNA sequencing should be inserted in editable format from the equation editor.

### Protein Expression and SDS-PAGE Analysis

The *P. pastoris* colonies were cultured in BMGY medium (5 mL tubes) at 28°C, 200 rpm until the OD_600_ of the culture reached to 4–6. The culture was then resuspended in BMMY medium (50 mL) containing YNB (1.34%) and biotin (4.0 μg/mL). Pure methanol was added to the culture to a final concentration of 1% every 24 h to maintain induction (Basit et al., 2018b). The induction process was done in triplicate.

The culture supernatants were pooled and analyzed by Tricine-SDS-PAGE (sodium dodecyl sulfate-polyacrylamide gel electrophoresis) followed by the purification through Ni-affinity column (Bio-Beads^TM^, Sweden) equilibrated with 1 × phosphate buffer (PB, 10 mM imidazole) (Basit et al., 2018b). Protein concentration was determined through the Bradford method by using bovine serum albumin as standard (Bio-Rad protein assay kit, Bio-Rad Laboratories, Inc.).

### Xylanase Activity Assay

Xylanase activity of the recombinant strains Mtxyn11A, Mtxyn11B, and Mtxyn11C was assayed by 3,5-dinitrosalicylic acid (DNS) method (Miller, 1959). The reaction system contained 1% BWX (w/v) in sodium phosphate buffer (pH 6.0) with appropriate diluted enzyme. The reaction system was then incubated at 50°C for 10 min, and terminated by the addition of 50 µL of NaOH (1 M). DNS reagent (150 µL) was added to the reaction mixture, boiled for 5 min, and then cooled at room temperature. Xylanase activity assay were performed in triplicate. One unit (U) of xylanase activity was defined as the amount of enzyme that released 1 µmol reducing sugar from the substrate (equivalent to xylose) per min under assay conditions.

The enzyme activity of the recombinant xylanase was compared with commercial xylanase from *Trichoderma longibrachiatum* (X2629, Sigma-Aldrich), under the assay conditions as described above. Optimum temperature and pH for commercial xylanase activity were sustained as per provided information.

### Expression of Xylanase in a Bioreactor

Fed-batch fermentation of xylanase was performed in a 7.5 L fermentor (Shanghai Boxing Bio-engineering Equipment Co., Ltd.) with 4 L basal salts medium (BSM) (glycerol (40 g/L), KOH (4.13 g/L), MgSO_4_ ·7H_2_ O (14.9 g/L), H_3_ PO_4_ (26.7 mL/L), CaSO_4_ ·7H_2_ O (14.9 g/L), K_2_ SO_4_ (18.2 g/L). BSM was supplemented containing 17.3 mL PTM1 solution. Fermentation of MYCTH_56237 and MYCTH_49824 was applied based on the *Pichia* Fermentation Process Guidelines (Invitrogen). The temperature and pH were maintained at 29°C and 5.0, respectively. Growth phase of the yeast cells were continued until the glycerol utilization from the feeding medium, which was indicated by the dissolved oxygen (DO) spike. When the glycerol was fully utilized from the feeding medium containing glycerol, 12 mL/L PTM1 solution was fed to fermenter according to a pre-adjusted DO level (10%). When the DO level reached higher than 60%, the glycerol feeding was stopped and pure methanol containing 12 mL/L PTM1 solution was pumped to induce the targeted gene expression, with the DO being set to 10–20%. Culture samples were collected at different time intervals to determine the OD_600_ and xylanase activity.

### Hydrolytic Activity of Xylanase

Hydrolytic activity of recombinant xylanase was estimated based on the hydrolysis of substrate BWX. For this purpose, reactions mixture containing 900 µL of 1% BWX (w/v) with 100 µL of appropriate diluted enzyme, in sodium phosphate buffer (pH 6.0) was prepared. The reaction mixture was then incubated at 50°C for 12 h and reducing sugars released from substrates were determined by DNS method (as above). The degradation rate (percent of total BWX) was calculated according to the following equation:

$$\mathrm{Degradation}\;\;\mathrm{rate}\;\left(\%\right)\;=\left(\frac{\mathrm{The}\;\mathrm{reducing}\;\mathrm{sugars}\;\mathrm{obtained}\;\mathrm{by}\;\mathrm{enzymatic}\;\mathrm{hydrolysis}\left(\mathrm{mg}\right)}{\mathrm{Amount}\;\mathrm{of}\;\mathrm{BWX}\;\mathrm{used}\left(\mathrm{mg}\right)}\right)\times 100\%\;$$ (1)

Hydrolysis products were analyzed by LC-20A HPLC (Shimadzu, Japan) with xylose and XOSs (X_5_, X_4_, X_3_, X_2_) as standards. HPLC system was equipped with a ROA-Organic Acid H^+^ (8%) column (Phenomenex) and a RIDL10A refractive index detector. The HPLC column was sustained at 50°C with H_2_SO_4_ (5 mM) as the mobile phase at a flow rate of 0.6 mL/min.

## Results and Discussion

### Improved Production of Xylanase

**FIGURE 2:**
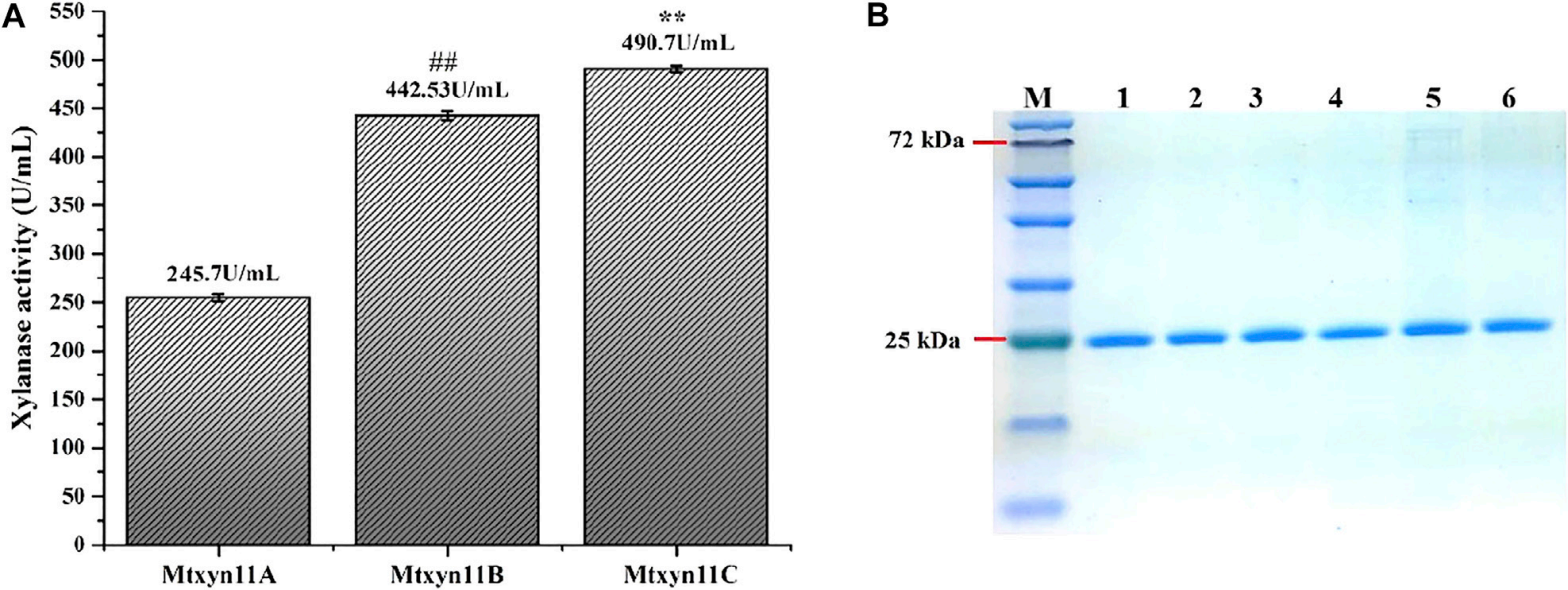
Enzyme activities and SDS-PAGE analysis of the recombinant proteins. **(A)** Schematic representation of the xylanase activity of each recombinant protein. Data represent mean ± SD of the independent triplicates. ** and ## indicates *p* < 0.05; **(B)** SDS-PAGE analysis of the purified recombinant proteins. M: Marker; Lane 1–2: Mtxyn11A; Lane 3–4: Mtxyn11B; Lane 5–6: Mtxyn11C.

In this study, we employed the secretion machinery system of *P. pastoris* and promising results were noted. Because of the secretion of few proteins, heterologous protein expression in secretary manner in *P. pastoris* is thought to be the foremost step in purification. The process was noted with key factors that played a tangible role in the secretion system. Therefore, xylanase activity of previously expressed MYCTH_56237 was further improved through adopting a commonly used strategy of replacement of α-factor in *P. pastoris* with signal peptide sequences. For this purpose, different expression plasmids Mtxyn11A (with native signal peptide of MYCTH_56237), Mtxyn11B (without native signal peptide of MYCTH_56237) and Mtxyn11C (without the sequence of α-MF) were constructed for the expression in *P. pastoris* (Figure 1). The expression plasmids were successfully cloned in the *P. pastoris* and positive transformants of recombinant strains Mtxyn11A, Mtxyn11B and Mtxyn11C were obtained on YPD agar plates containing Zeocin (100 μg/mL) and further regulated by an AOX1 promoter (induced by methanol). After 120 h of cultivation in BMMY medium, significantly observed was the parameter of a single peptide involved in the secretion. The xylanase activity of the recombinant strain Mtxyn11C (with native signal peptide) was significantly 1.2-folds higher than that of Mtxyn11B (without native signal peptide), followed by Mtxyn11C (Figure 2A). The SDS-PAGE analysis of the recombinant proteins (Mtxyn11A, Mtxyn11B and Mtxyn11C) indicated the estimated molecular weights of about 26 kDa, almost near to the range of calculated weights of MYCTH_56237 (Figure 2B). The single peptide is found to guide protein trafficking through the secretory pathway. The most common signal peptides which are integrated in plasmid of *P. pastoris* include *S. cerevisiae* α-MF, which have been used efficiently for the secretion of various heterologous proteins in *P. pastoris* (Kommoju et al., 2007). Protein secretion in yeast follows a pathway from endoplasmic reticulum to Golgi and then tracks to extracellular space. An important step in this process is site-specific breakage of the signal peptide from pre-protein (Lee et al., 2003). Subsequently, the sequences of signal peptide were evaluated to improved protein expression levels in *P. pastoris*, with a special focus on secretion efficiency of the α-factor signal peptide (Huang et al., 2014). The variations in research findings have been reported indicating conflicts in results. Reportedly these conflicting results have been reported in some studies conducted by different researchers (Ghosalkar et al., 2008).

**FIGURE 3:**
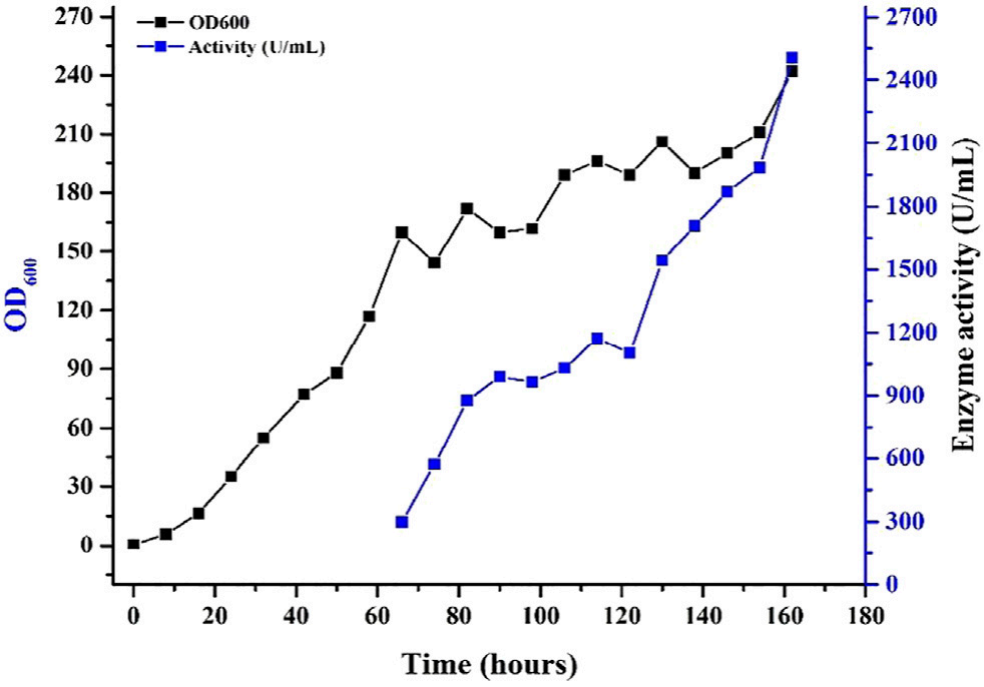
Expression of the recombinant Mtxyn11C in a 7.5 L fermentor for xylanase production.

The present study also showed that replacement of α-MF secretion signal with native signal sequence of xylanase, led to an enormously higher yield of xylanase in the culture supernatant in shake flasks and in 7.5 L fermentor as well. Fed-batch fermentation of the (improved activity of xylanase) Mtxyn11C was employed in 7.5 L fermentor. The recombinant strain (*Mtxyn11C*) was inoculated to the medium and glycerol was supplemented after 24 h. The rate of dissolved oxygen (DO) was raised up to 50% after the termination of glycerol feeding. Methanol was then pumped into the medium to induce the xylanase production with a stepwise increasing rate. Xylanase activity in the culture supernatant was not detected until the initiation of methanol feeding. Followed by the methanol feeding for 83 h, the maximum xylanase activity in the supernatant was 2503 U/mL (Figure 3). Xylanase activity of Mtxyn11C was successfully improved from 2,010.4 to 2503 U/mL, which is significantly higher than the native MYCTH_56237 (Basit et al., 2018b).

### Hydrolytic Activity of Mtxyn11C and Applications

**FIGURE 4:**
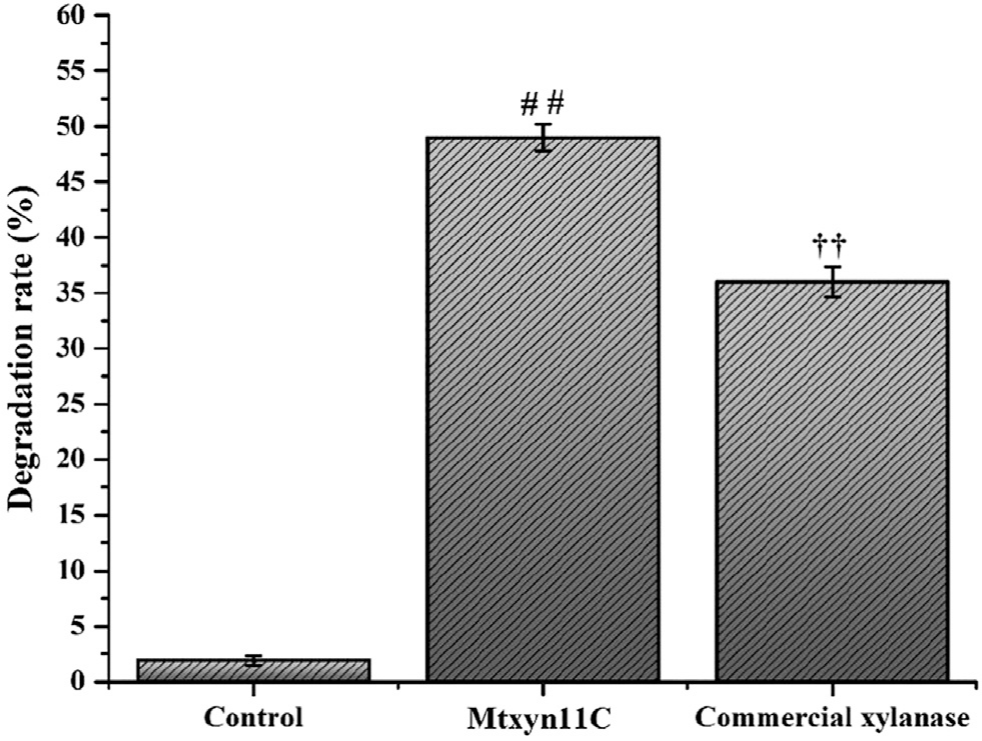
Degradation efficiency of Mtxyn11C and commercial xylanase towards BWX. Data represent mean ± SD of the independent triplicates. ^# #,††^ indicates *p* < 0.05.

**FIGURE 5:**
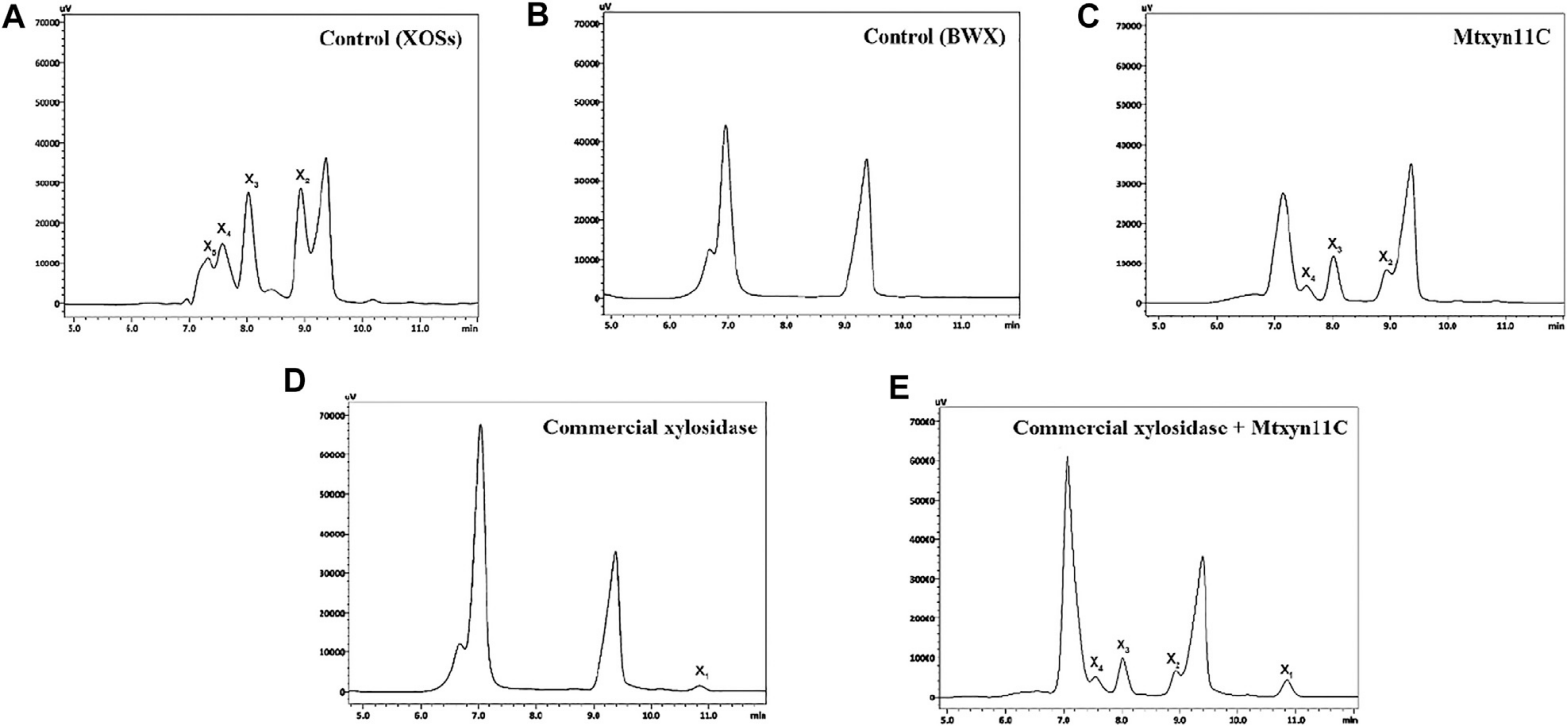
HPLC profile of hydrolyzed products of BWX by Mtxyn11C. **(A)** Control for the standards (XOS); **(B)** Control for BWX without adding an enzyme; **(C)** Degradation of BWX by Mtxyn11C (X_1_, xylose; X_2_, xylobiose; X_3_, xylotriose; X_4_, xylotetrose); **(D)** Degradation of BWX by commercial β-xylosidase; **(E)** Degradation of BWX by the combine action of Mtxyn11C and commercial β-xylosidase.

Production of xylooligosaccharides is great research interest due to its importance in various industrial sectors, in biofuel production and formation of the other valuable biomolecules. Therefore, the hydrolytic mode of action was estimated by inoculating Mtxyn11C (2 U) with 1% BWX, in sodium phosphate buffer (pH 6.0) at 50°C. The maximum degradation rate of Mtxyn11C was observed towards BWX and reached to 49% in 6 h (Figure 4). Moreover, HPLC analyses were performed and it was observed that a significant amount of xylobiose (X_2_), xylotriose (X_3_) and xylotetrose (X_4_), among the hydrolysis product (Figures 5A–C) was present. In comparison, the degradation efficiency of Mtxyn11C against the commercial endoxylanase (2 U) from *Trichoderma longibrachiatum* (according to conditions provided by the manufacturer’s) and Mtxyn11C was observed more effective than commercial endoxylanase (Figure 4).

Furthermore, the complete degradation of lignocellulosic biomass entails the specific degrading enzymes acting synergistically and considered as the rate-limiting step in biofuels production. Therefore, in this study, degradation efficiency of Mtxyn11C was evaluated with that of synergistically acting commercial β-xylosidase. The degradation efficiency of Mtxyn11C was further examined with that of synergistically acting commercial β-xylosidase (1.6 U/20 mg) using BWX as substrate (in pH 6.0 buffer at 50°C for 1 h), using identical reaction conditions. Consequently, Mtxyn11C liberated a notable amount of xylose, with a trace amount of XOSs (X_2_, X_3_, X_4_). The amount of xylose produced in result of both enzymes synergism was higher than that of commercial β-xylosidase (Figures 5D,E). Mtxyn11C was obviously more effective for BWX degradation and liberated a notable amount of xylose, with a trace amount of XOSs (X_2_, X_3_, X_4_). The oligosaccharides (X_2_, X_3_, X_4_) are used in dietary supplements and used as byproducts in various chemical industries (Chapla et al., 2012; Rastall, 2010).

**FIGURE 6:**
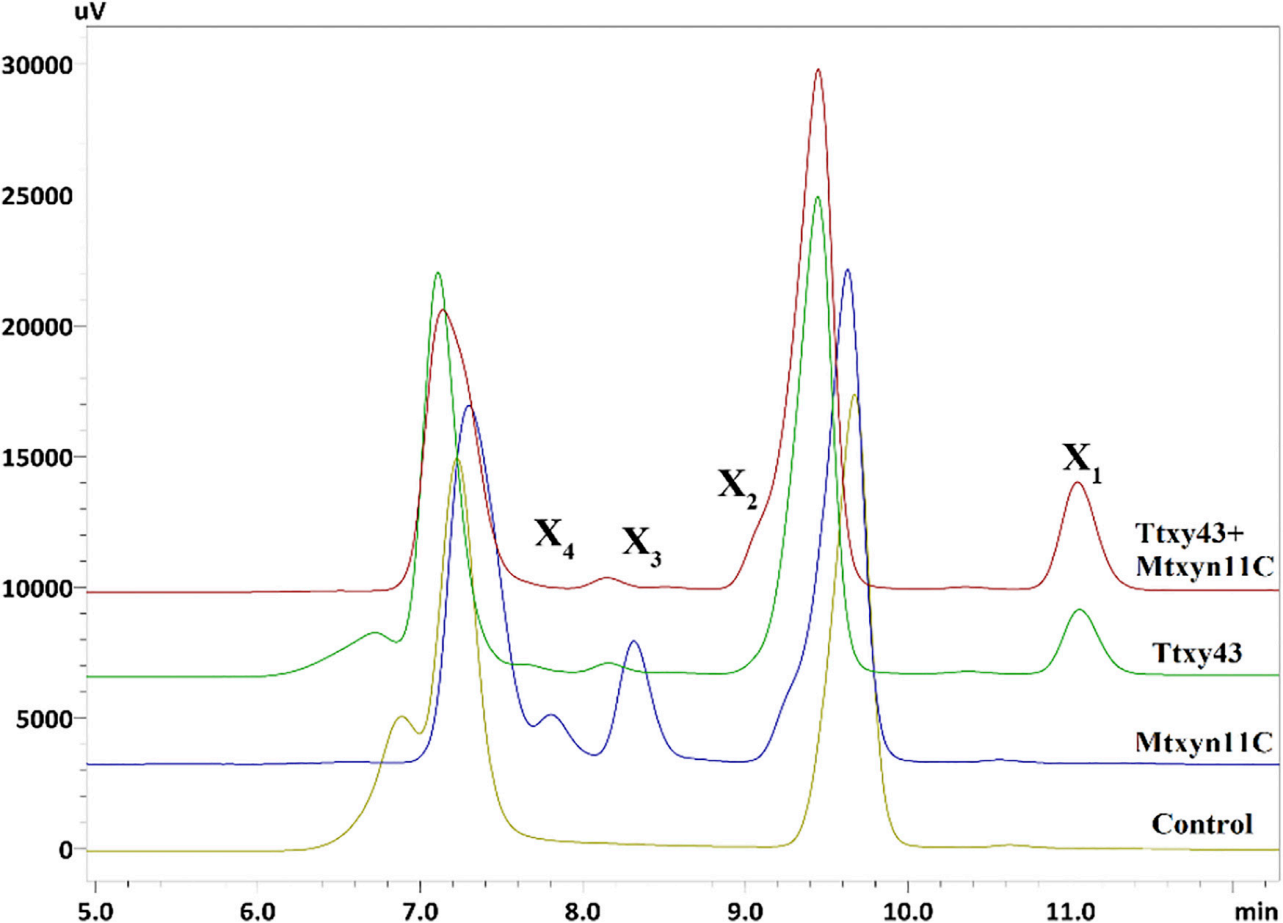
HPLC profile of hydrolyzed products of BWX, showing hydrolytic efficiency of Mtxyn11C and Ttxy43 for conversion of BWX. Control: BWX without added enzyme. (X_1_, xylose; X_2_, xylobiose; X_3_, xylotriose; X_4_, xylotetrose).

The synergic study of Mtxyn11C was performed against a multifunctional xylanolytic enzyme from *M. thermophila* (termed Ttxy43). Ttxy43 showed multi-enzyme activities of endoxylanase (105.42 U/mg), β-xylosidase (80.8 U/mg) and α-L-arabinofuranosidase (15.81 U/mg). The catalytic mode of action of Ttxy43 towards BWX revealed that xylanase firstly degrades xylan to XOSs (X_2_, X_3_, X_4_) as intermediates, which were then rapidly hydrolyzed into single xylose by β-xylosidase [14]. In order to improve further the xylose production, 2 U of Mtxyn11C and Ttxt43 was incubated with 1% BWX in sodium phosphate buffer (pH 6.0) at 50°C for 1 h. Consequently, the xylose production was successfully improved by 40% in short duration of 30 min (Figure 6). This improved conversion of xylan into xylose along with the production of X_2_, X_3_ and X_4_, is a highly desirable property with the potential to reduce cost and energy required for the production of pure xylose. Therefore, these findings elucidate an effective integrated degradation mechanism which provides innovative approaches for biomass energy or biofuels production.

## Conclusion

In this study, the xylanase activity from previously identified from the *M. thermophila* was further increased through construction of engineering strains of *P. pastoris* by substituting of the secretory α-MF with the native signal peptide. The xylanase activity was significantly enhanced by 1.2-folds compared with the native one. The hydrolytic activity of MTxyn11C towards the BWX was further revealed the production of xylobiose, xylotriose and xylotetrose from BWX. This study also elucidated the improved conversion of xylan into xylose (by 40%) when integrated with the multifunctional xylanloytic enzyme. Altogether, these findings illustrate innovative strategies for biomass energy or biofuels production.

## Data Availability

The raw data supporting the conclusion of this article will be made available by the authors, without undue reservation.
