# Supplementary material for: Improved Production of Xylanase in Pichia pastoris and Its Application in Xylose Production From Xylan
Source: Front Bioeng Biotechnol. 2021 Aug 27;9:690702. doi: 10.3389/fbioe.2021.690702 (PMC8429496; doi:10.3389/fbioe.2021.690702)
Supplement: Supplementary file 1 [file Table1.DOCX]

Supplementary Material

Improved production of xylanase in *Pichia pastoris* and its application in xylose production from xylan

**Ting Miao^1^, Abdul Basit^1,2^*, Junquan Liu^1^, Fengzhen Zheng^1^, Kashif Rahim^3^, Huiqiang Lou^1^ and Wei Jiang^1^***

^1^ State Key Laboratory of Agro-Biotechnology, College of Biological Sciences, China Agricultural University, Beijing 100193, China.

^2^Department of Microbiology and Molecular Genetics, Faculty of Life Sciences, University of Okara, 56130, Pakistan.

^3^Department of Microbiology, Cholistan University of Veterinary and Animal Sciences (CUVAS), Punjab, Bahawalpur 63100, Pakistan.

*** Correspondence:**

**Abdul Basit**, E-mail: abdul_9090@yahoo.com

**Wei Jiang**, E-mail: jiangwei01@cau.edu.cn, Tel: 8610-62731440

# Supplementary Table

# Table S1: Primers used for study

| **Primer** | **Sequence** | **Sites** |
| --- | --- | --- |
| MYC_56237-F | AATTCTTCCCATTCAACGCTACTCA | ***EcoR*I**  ***Xba*I** |
| MYC_56237-R | TCTAGAAGCTTGAACAGTGATAGAAG |  |
| AOX- F | GACTGG TTCCAATTGACAAGC |  |
| AOX- R | GCAAATGGCATTCTGACATCC |  |
| Mtxyn11C-F | ACAACTAATTATTCGAAACGATGGTTTCTGTTTCTGTTAACTGT | ***EcoR*I**  ***Xba*I** |
| Mtxyn11C-R | ACAGCCTTAACAGAAACCATCGTTTCGAATAATAATTTAGTTGT |  |
| Mtxyn11B*-*F | ACAACTAATTATTCGAAACGTTCCCCTTCAACGCTACTCA | ***EcoR*I**  ***Xba*I** |
| Mtxyn11B*-*R | TGAGTAGCGTTGAAGGGGAAGGGGAACGTTTCGTTTCGAATAATTAGTTGT |  |
| Mtxyn11A*-*F | AGAAAAGAGAGGCTGAAGCTTTCCCCTTCAACGCTACTCA | ***EcoR*I**  ***Xba*I** |
| Mtxyn11A*-*R | TGAGTAGCGTTGAAGGGGAAAGCTTCAGCCTCTCTCTTTTCT |  |
